# Maternal Health Indicators Among Migrant Women Construction Workers

**DOI:** 10.4103/0970-0218.43240

**Published:** 2008-10

**Authors:** Amrit Abrol, Meenu Kalia, BP Gupta, AS Sekhon

**Affiliations:** Department of Community Medicine, Gian Sagar Medical College and Hospital, Ramnagar, Banur, Punjab, India. E-mail: minumansharma@yahoo.com

Sir,

More than half a million women die annually worldwide because of pregnancy-related complications.(1) About 90–95% of these women come from developing countries.(2) The maternal mortality ratio in India is 407 per 100,000 live births (NFHS-3).(3) The Millenium Development Goal 5 aims at reducing the maternal mortality ratio by three quarters.(4)

This paper studies the utilization of antenatal care services along with breastfeeding practices among migrant women construction workers as an indicator of their health status. This study was conducted among migrant construction workers employed at the Gian Sagar Medical College and Hospital Construction site, Banur, District Patiala, Punjab.

This cross-sectional study was completed among female construction workers during August–October 2007. There were 564 females working at the construction site. Out of these, 430 females were in the reproductive age group and 308 females had delivered in the last 1 year. All 308 females were included in this study. A team of female health workers and a Medical Officer obtained information regarding antenatal care, place of delivery, and breastfeeding practices with respect to the previous pregnancy by using a pre-designed and pre-tested proforma. The height and weight of each study subject was measured using standardized equipment and pallor was assessed clinically by the accompanying medical officer. Collected data was analyzed and compared with national and state figures.

Among the studied population, 64.4% of females were illiterate [Table 1]. The majority of the literate females were educated only up to the primary level.

**Table 1:** Key reproductive and child health indicators

Key indicators	Present study (%)	Indian NFHS-3 (%)	Punjab NFHS-3 (2005–06) (%)
Female illiteracy	64.4	59.1	47.3
Women with BMI below normal	58.8	38.8	14.5
Mothers who had at least 3 ANC visits for the last birth	10.5	42.8	70.2
Mothers who consumed IFA for 90 days or more during their last pregnancy	9.7	18.1	24.2
Institutional birth	15.0	31.1	48.4
Delivery by a trained birth attendant	18.5	39.1	67.4
Initiation of breastfeeding within the first hour	44.9	21.5	7.3
Exclusive breastfeeding (0–6 months)	48.5	48.3	32.6
Complementary feeding (6–9 months)	74.1	53.8	50.0

ANC = Antenatal care; BMI = Body mass index; IFA = Iron and folic acid

Clinical anemia among never married women was found to be 54.9%. Only 10.5% of the study subjects had received 3 or >3 antenatal care check-ups during their last pregnancy and 9.7% had consumed tablets for iron and folic acid for 90 days or more. In this study, 64.7% were unprotected against tetanus.

Of these women, 15% had institutional deliveries and only 18.5% of those who had delivered at home were attended to by trained birth attendants. The breastfeeding practices were found to be much better as initiation of breast feeding within the first hour was practiced by 44.9% of the mothers and 48.5% of them practiced exclusive breast feeding for 6 months. These figures are comparable with the other studies conducted in Chandigarh(5) and Maharashtra.(6)

This study depicts the wide disparity in maternal and child health indicators in this population in comparison with the national and state averages. This information will be useful for local administrators to effectively plan the coverage strategies for this population.
